# The effect of varus rearfoot wedges on hallux dorsiflexion resistance

**DOI:** 10.1186/s12891-024-07182-x

**Published:** 2024-01-22

**Authors:** Álvaro Gómez-Carrión, José Manuel Reguera-Medina, Ignacio Ayerra-Andueza, Juan Francisco Cortés-Morán, Alfonso Martínez-Nova, Rubén Sánchez-Gómez

**Affiliations:** 1 Nursing Department, Faculty of Nursing, Physiotherapy, and Podiatry, Universidad Complutense de Madrid, Madrid, 28040 Spain; 2 Clinical Sanipie, Utrera, Sevilla, 41704 Spain; 3 Clinical Ayerra, Almeria, 04001 Spain; 4 Nursing Department, Universidad de Extremadura, Plasencia, 10600 Spain; 5 IdISSC, Institute for Health Research, Hospital Clínico San Carlos, Madrid, 28040 Spain

**Keywords:** Metatarsophalangeal joint, Proximal phalanx, Resistance, Force, Jack´s Test, Varus rearfoot wedges

## Abstract

**Background:**

The first metatarsophalangeal joint (MTPJ), which includes the first metatarsal and proximal phalanx, plays a crucial role in gait and impacts the windlass mechanism. Disruptions to this mechanism are implicated in various foot pathologies. Jack’s Test serves as a valuable tool for clinicians to assess the functionality of the MTPJ. Varus rearfoot wedges (VRFWs) are a common treatment employed in the management of lower limb pathologies. The impact of VRFWs on the resistance of the first MTPJ during Jack´s Test is currently unknown. This study aimed to measure the influence of VRFWs on the resistance of the first MTPJ during Jack´s Test. The secondary objective was to validate a new measurement method using a digital force gauge.

**Methods:**

Thirty participants (17 women and 13 men) were enrolled. A digital force gauge measured the weight-bearing force needed for Jack’s Test, thereby evaluating the effects of VRFWs of different angulations. The Kolmogorov–Smirnov test confirmed that the data followed a normal distribution (*p* > 0.05). The nonparametric Friedman test (*p* < 0.001) showed that there were significant differences among all VRFWs, while the Wilcoxon test (*p* < 0.001) showed that there were differences between barefoot conditions and 3°, 5°, and 8° VRFWs. Results: The use of 8° VRFWs yielded a statistically significant reduction in the passive dorsiflexion force of hallux during Jack’s Test (12.51 *N* ± 4.12, *p* < 0.001).

**Conclusions:**

The use of VRFWs has been observed to reduce dorsiflexion resistance in the proximal phalanx of the first MTPJ during Jack’s Test. Additionally, the digital force gauge was proven to be a valid tool for conducting Jack’s Test, thus offering a reliable measurement method.

## Introduction

The first metatarsophalangeal joint (MTPJ) comprises the head of the first metatarsal and the proximal phalanx of the hallux [1]. Dorsiflexion of the first MTPJ is crucial during the push-off phase of the normal gait cycle in the sagittal plane [2, 3]. This joint experiences significant biomechanical demand, with a load force ranging from 40–60% of body weight during normal walking, and this load increases by 800% during sports activities [4]. The average range of motion for this joint is 30°-50° of dorsiflexion motion depending on the foot model used in gait analysis [5]. It has been suggested that a decrease in dorsiflexion movement of the first MTPJ can alter foot function, potentially leading to an inefficient gait during weight transfer in the push-off phase [6]. An inefficient gait while walking thousands of steps daily may cause a biomechanical alteration of the lower limb that could be related to chronic back, hip, knee and forefoot pain [7–9]. The first MTPJ region is a common site of injury among athletes engaged in jumping and running. Research by Rodeo [10] revealed that 45% of American football players sustained a first MTPJ injury during their professional careers.

The windlass mechanism is formed by the plantar fascia, whose origin is anchored to the medial tuberosity of the calcaneus and extends along the foot to the five toes. The distal insertion takes place in the proximal phalanx of each toe, with the most significant being at the first MTPJ [11–13]. When the phalanges undergo dorsiflexion, they induce tension in the plantar fascia, resulting in a plantar flexion moment of the metatarsal heads. This leads to an elevation in the height of the medial arch of the foot, an inversion moment of the calcaneus and external rotation of the tibia. Research has suggested that this mechanism is involved during the push-off phase of the gait cycle [14, 15].

Jack’s Test (Hubscher’s Test) is a broad clinical manoeuvre employed by podiatrists, orthopaedists and physicians to assess the function of the first MTPJ. This test involves the passive dorsiflexion of the first MTJP with the individual in a relaxed stance while elevating the height of the medial arch. Clinicians utilize this test to evaluate the force needed for passive dorsiflexion of the hallux at the first MTPJ. A minimum dorsiflexion force of the hallux with a concurrent increase in the height of the medial arch is considered normal. Nevertheless, there is controversy regarding whether this manoeuvre holds predictive value during walking [16, 17]. Recent studies propose that assessing joint force is superior to evaluating the range of motion in relation to gait analyses [18, 19].

The varus rearfoot wedge (VRFW) is commonly employed in the management of lower limb pathologies, including patellofemoral pain syndrome [20], medial tibial stress syndrome [21], posterior tibial dysfunction [22] and plantar fasciitis [23]. It has been proposed as a treatment for the management of foot problems during running [24]. Assessing the force needed for passive dorsiflexion of the hallux at the first MTJP provides clinicians with kinetic information on the windlass mechanism. For instance, this test may enable clinicians to gauge the potential effectiveness of foot orthosis by evaluating the decrease in passive dorsiflexion force of the hallux with treatment.

The impact of VRFWs on the resistance of the first MTPJ during Jack´s Test is currently unknown. This study aimed to measure the influence of VRFWs on the resistance of the first MTPJ during Jack´s Test. The secondary objective was to validate a new measurement method using a digital force gauge. The hypothesis posited in this study suggests that VRFWs lead to a decrease in passive resistance during dorsiflexion of the hallux at the first MTPJ, as measured by the digital force gauge.

## Materials and methods

The study received approval from the Bioethics and Biosafety Committee of the University of Extremadura (CBBUEx) under the code ID: 15-06-2023//89_2023. The ethical and human criteria established in the Declaration of Helsinki were followed. All participants were informed of the necessity to sign the informed consent form in accordance with Organic Law 15/99 of 13 December.

### Participants

The sample size calculation for this study was conducted by the Calculation Center of the Complutense University of Madrid. The study assessed differences in dorsiflexion resistance during Jack´s Test with the use of different VRFWs. Adherence to guidelines and regulations was in accordance with Strengthening the Reporting of Observational Studies in Epidemiology (STROBE) for this study. The sample size was determined using G*power software (version 3.1.9.6, Kiel University, Kiel, Germany) with a power level of 80%, considering a confidence interval (CI) of 95%, alpha level of 0.05, beta level of 20% and accounting for a drop rate of 20%. A simple sample size of 30 subjects (*n* = 17 women and *n* = 13 men) was deemed necessary. A convenience sample of 45 subjects was chosen to address potential common participant losses.

The inclusion criteria were as follows: 1) subjects aged between 18 and 65 years of both sexes [25]; 2) a range of motion greater than 30° of dorsiflexion of the first MTPJ as measured with a goniometer [26, 27]; 3) a Foot Posture Index (FPI) between 0 and + 5 [28, 29]; and 4) no previous foot injury in the last 12 months [30]. The exclusion criteria were as follows: 1) a history of previous surgery in the first MTPJ or foot alterations, such as hallux valgus or rigidus, and 2) diagnosis of neurological problems affecting balance [25].

### Instruments, measurement procedures, and variables

Subjects were assessed for adherence to the inclusion criteria through a face-to-face interview conducted by a member of the research team. The entire procedure was explained, and upon participation, subjects were requested to read and sign the informed consent form. Subsequently, demographic data (age, height, weight, previous lower limb injury) were collected.

**Fig. 1:**
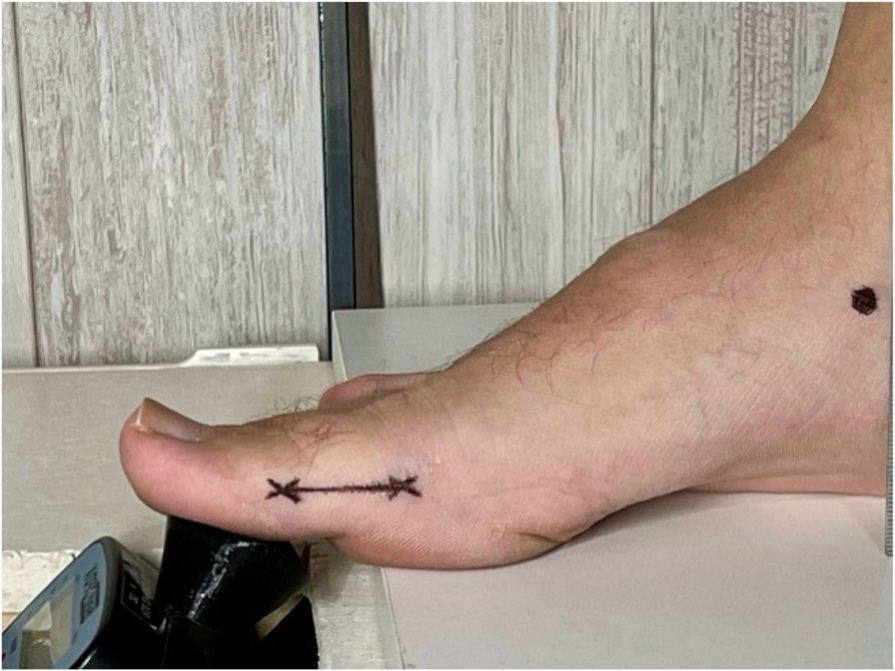
The digital force gauge and a lever arm situated at the proximal phalanx

The subject was positioned lying on the examining table in the supine position. The total range of motion of the first MTPJ was measured using a classical goniometer [17]. The distal medial tuberosity of the proximal phalanx of the hallux was marked with a demographic pencil, considering this point as the moment arm for the hallux [18] (Fig. 1). Following this, the FPI was measured as an established and valid tool widely used in foot cataloguing in numerous studies. The researcher then identified the medial tuberosity of the navicular and marked it with the demographic pencil, following the procedure outlined in Moisan’s research [28].

**Fig. 2:**
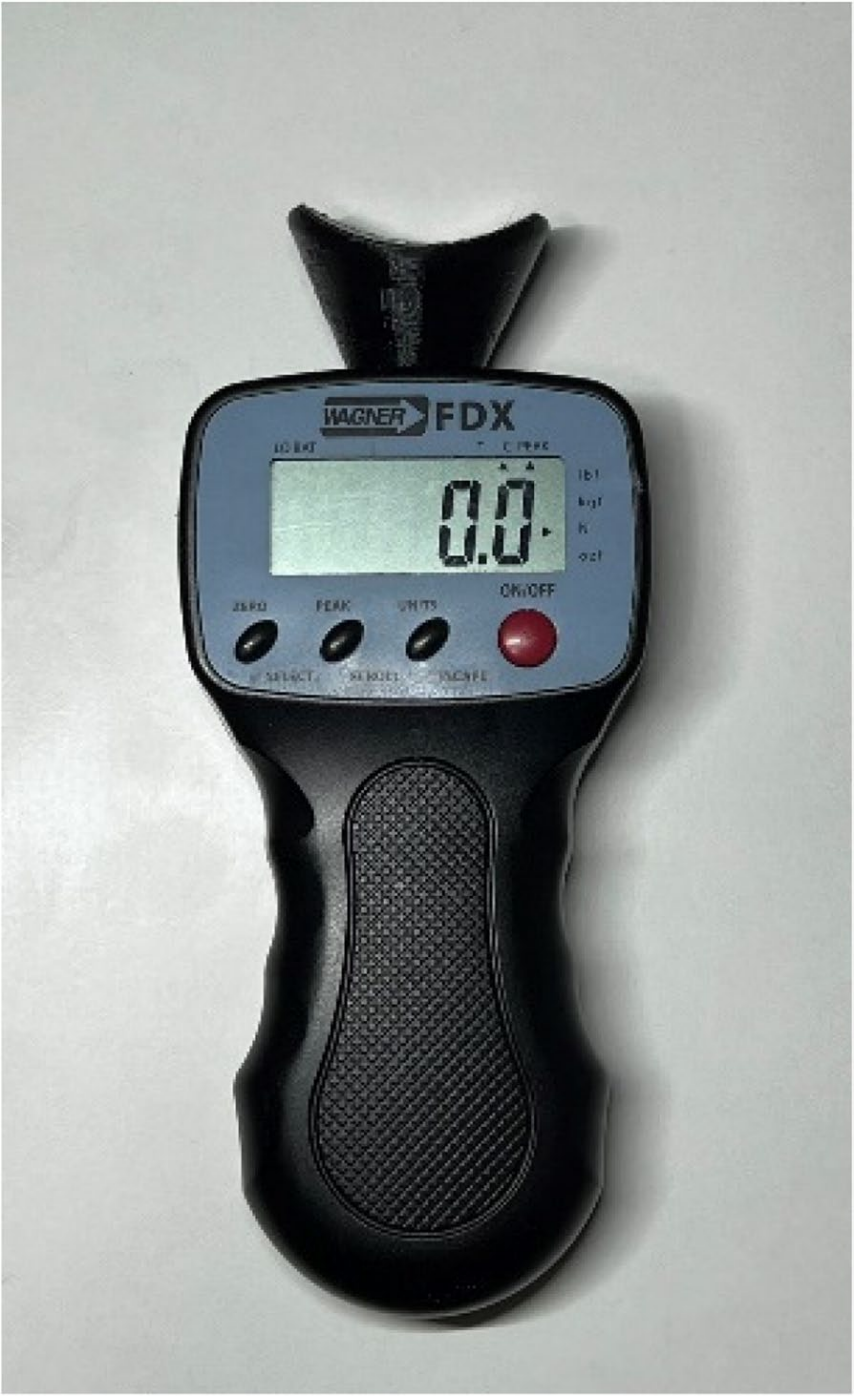
Digital force gauge with an adapter for the proximal phalanx of the first MTJP

**Fig. 3:**
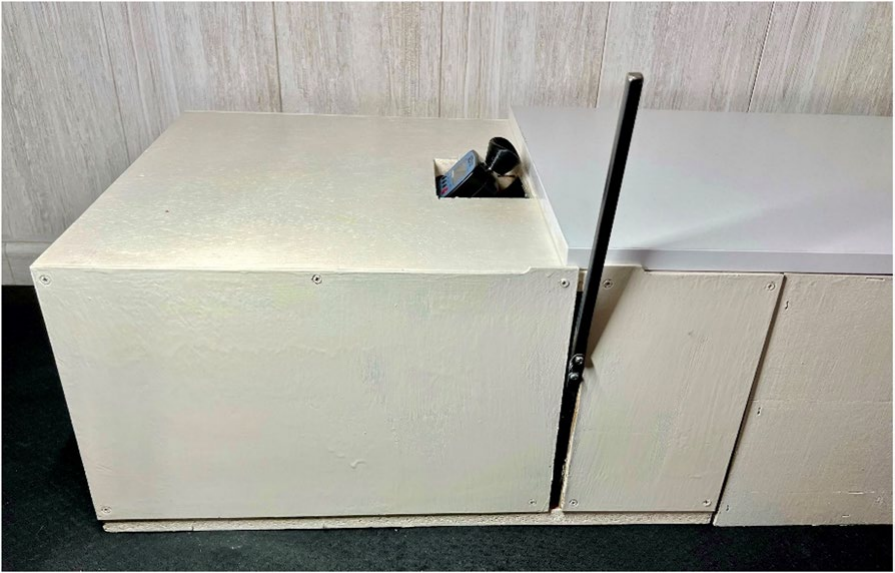
New device with the digital force gauge formed by a pulley and lever system

**Fig. 4:**
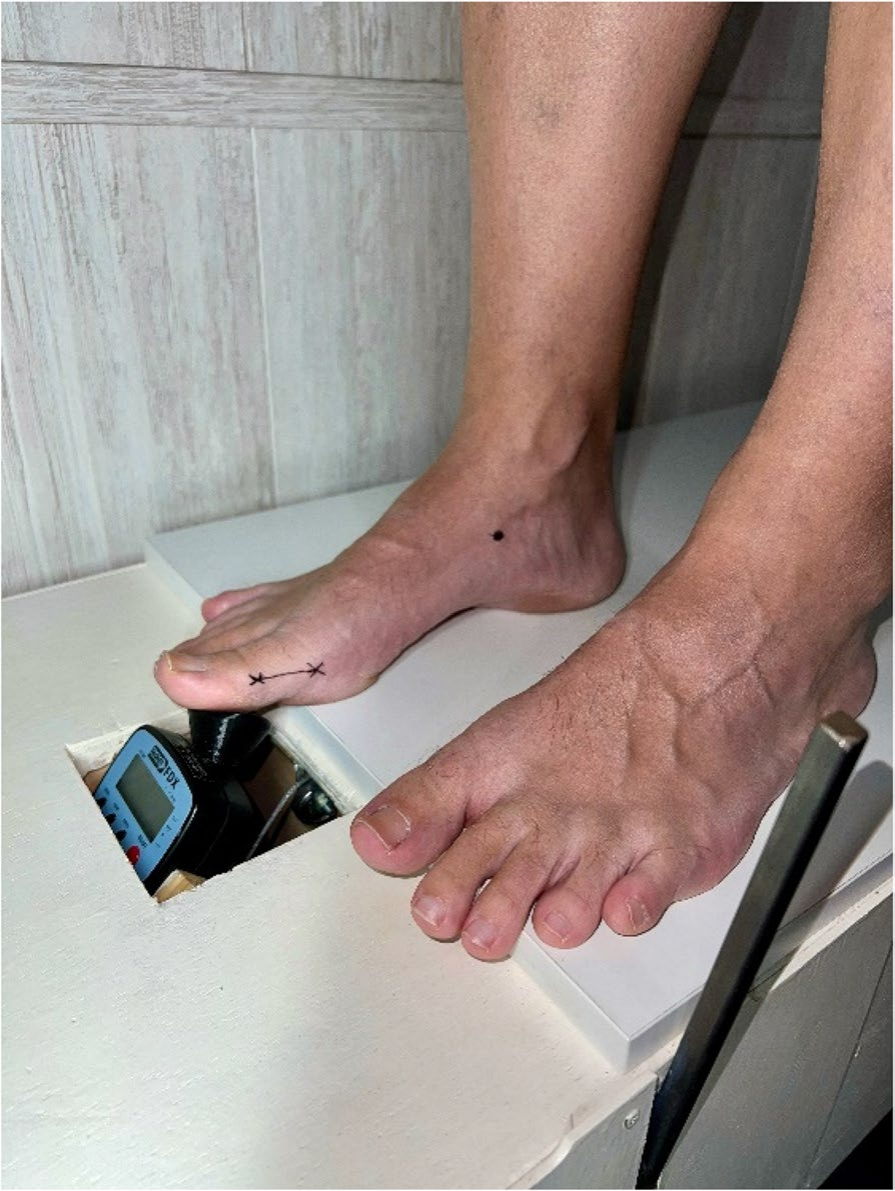
The subject with the digital force gauge in a relaxed stande position

**Fig. 5:**
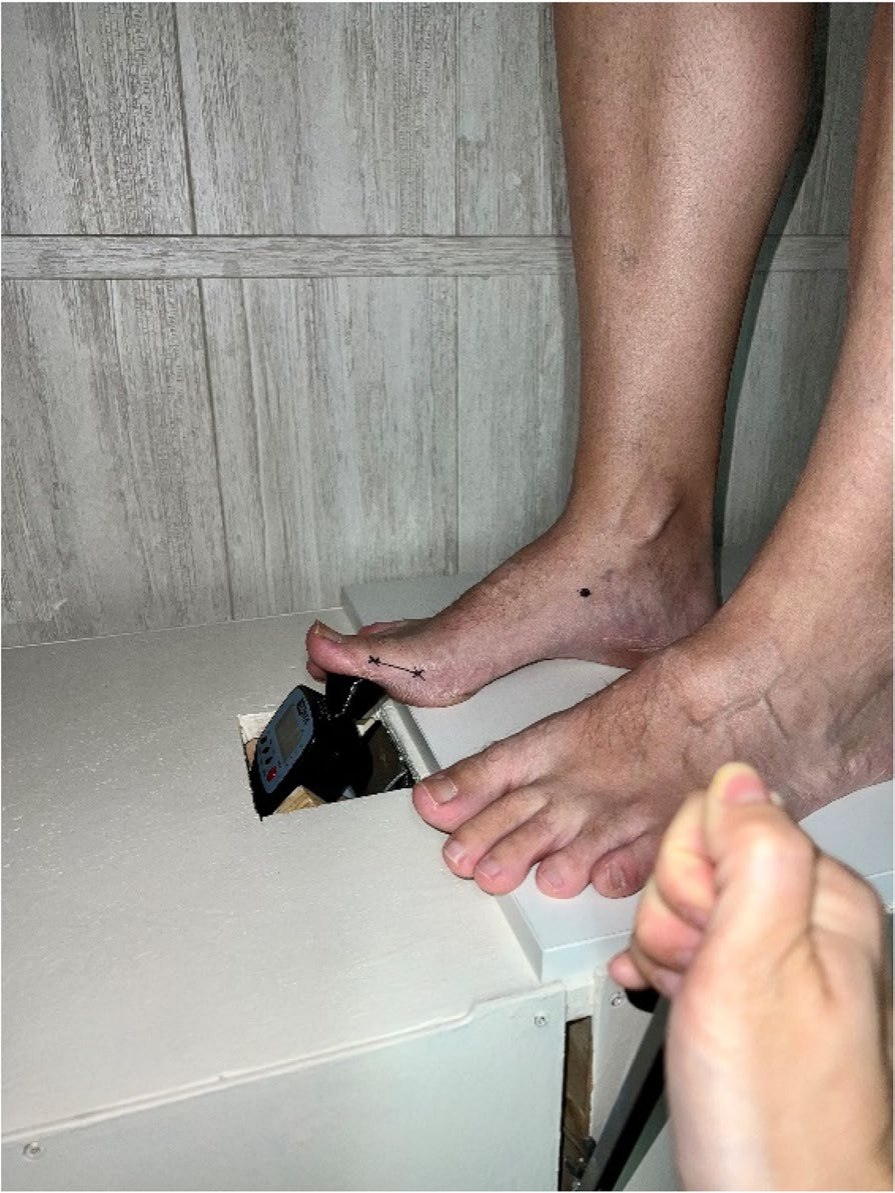
Researcher pulling the lever and quantifying the force with the digital force gauge

The subject assumed a relaxed stance on the new device, and the researcher positioned the digital force gauge adapter using the mark on the proximal phalanx. Measurements were conducted with a digital force gauge (FPX®25, Wagner Instruments®®, Greenwich, CT, USA) (Fig. 2). This digital force gauge boasts a precision of ± 0.3% of the full scale and has been utilized in various previous research studies [31–33]. Capable of measuring force in Newtons, pound-force, kilograms-force and, ounce-force, our study utilized Newtons as the unit of force. The digital force gauge was placed on a piston of rectilinear motion at a 45° angle perpendicular to the proximal phalanx of the first toe [34] (Fig. 3). The objective was to replicate the performance of Jack’ test, employing the digital force gauge and a lever arm situated at the proximal phalanx. The upwards scrolling of the digital force gauge was facilitated by a pulley and lever system to induce a passive dorsiflexion movement of the hallux (Figs. 4 and 5). This methodology enables the quantification of force in Newtons needed for passive dorsiflexion of hallux during Jack’s Test. The proximal phalanx displacement and joint stiffness were not measured. We followed the methodology outlined in Moisan’s research [28] to ascertain the optimal timing for assessing hallux resistance. By marking the medial tuberosity of the navicular, our researcher identified an elevation in the medial arch height. Unlike the original research, our investigator employed a ruler to quantify the arc height change, rather than relying solely on visual observation.

**Fig. 6:**
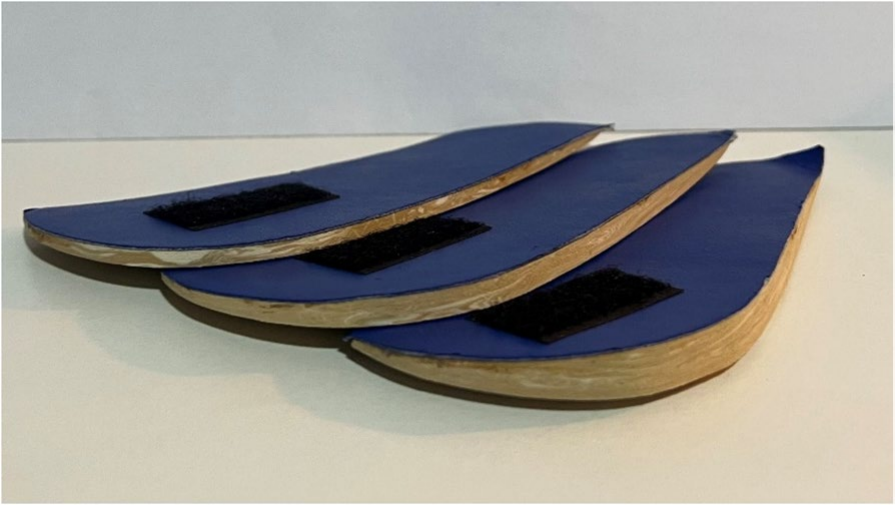
Varus rearfoot wedges of 3°, 5° and 8°

Two researchers performed the measurements. Each condition, barefoot and with VRFWs, was measured three times by each researcher, for a total of six repetitions for each condition. In this study, three VRFWs of 3, 5 and 8 degrees were used randomly to mitigate the order effect [35]. There was a 10-second break between each measurement. VRFWs were placed by the first researcher (A.G.C.) with more than ten years of experience. They were manufactured from 70 shore of ethylene vinyl acetate (EVA) with a length of 10 cm and a width of 5 cm (Fig. 6). All the VRFWs had the same colour and were used randomly. VRFWs were placed on both feet to prevent instability, and the measurement of the dominant foot was recorded. The dominant foot was determined by asking participants which leg they used to kick a ball.

### Statistical analysis

The statistical unit of the Complutense University of Madrid used SPSS Version 20.0 for Windows (IBM Corp., Armonk, NY, USA) to determine whether the sample followed a normal distribution. The Kolmogorov–Smirnov test determined that the sample distribution was normal (*p* > 0.05). Two tests for *p* values for multiple samples were used. The nonparametric Friedman test (*p* < 0.001) was used to analyse whether there were significant differences among all VRFWs, and the Wilcoxon test (*p* < 0.001) was used to determine whether there were significant differences between barefoot and VRFWs of 3°, 5°, and 8°.

## Results

The initial sample comprised 30 subjects (13 men and 17 women); however, due to typical sample loss, 45 subjects were initially selected. Thirteen subjects did not meet the inclusion criteria, and two withdrew from the study during its course. Table 1 shows the sociodemographic characteristics of the sample.

**Table 1:** Summary sociodemographic measurements

	N	Mean	SD	Minimum	Maximum
**Age (Years)**	30	42.37	14.21	18	64
**Weight (kg)**	30	64.10	10.5	46	85
**Height (cm)**	30	166.42	7.11	156	179
**FPI (Scores)**	30	2.00	1.24	0	5

*Abbreviations:* *SD* Standard deviation, *FPI* Foot posture index

**Fig. 7:**
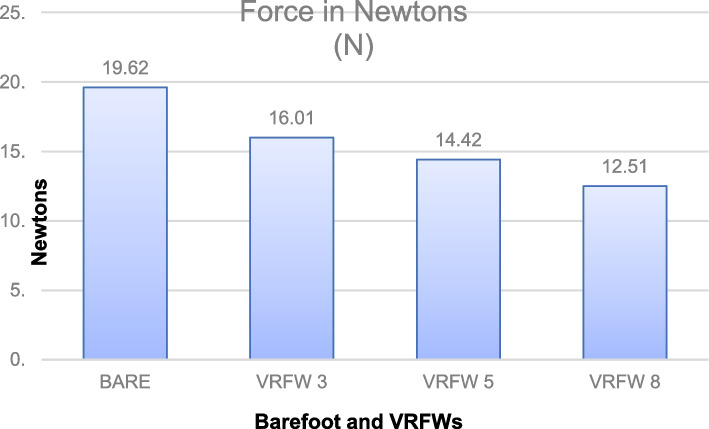
The graph shows the hallux dorsiflexion resistance in Newtons with VRFWs and barefoot. BARE = barefoot; VRFW3 = Varus rearfoot wedge 3°; VRFW5 = Varus rearfoot wedge 5°; VRFW8 = Varus rearfoot wedge 8°

The reproducibility of the data was determined with the intraclass correlation coefficients (ICCs), standard error of measurement (SEM), and minimum detectable change (MDC) of the different conditions (barefoot and the three VRFWs), as shown in Table 2. The ICC for intratester reliability had a range of 0.989–0.998, and the ICC for intertester reliability had a range of 0.960–0.973. These results, according to the Landis and Koch classification, indicate perfect reliability [36]. The results in Newtons for dorsiflexion of the proximal phalanx of the first toe in each condition are shown in Table 3 and Fig. 7. The force needed in Newtons to move the dorsiflexion of the proximal phalanx of the first toe was less with the use of VRFWs and decreased with increasing thickness of the wedges (*p* < 0.001). With a VRFW of 8°, the force in Newtons needed to move the dorsiflexion of the proximal phalanx of the first toe was the lowest at 12.51 ± 4.12 N (*p* < 0.001).

**Table 2:** The reliability of data for ICCs, SEM, and MDC in Newtons for each situation

**Variable**s	BARE					VRFW3					VRFW5					VRFW8				
	**SD**	**ICC Intra (95% CI)**	**ICC Inter (95% CI)**	**SEM**	**MDC**	**SD**	**ICC Intra (95% CI)**	**ICC Inter (95% CI)**	**SEM**	**MDC**	**SD**	**ICC Intra (95% CI)**	**ICC Inter (95% CI)**	**SEM**	**MDC**	**SD**	**ICC Intra (95% CI)**	**ICC Inter (95% CI)**	**SEM**	**MDC**
Newtons (N)	5.18	0.995 (0.992–0.998)	0.971	0.35	0.96	4.09	0.994 (0.989–0.997)	0.960	0.32	0.88	3.98	0.995 (0.990–0.998)	0.965	0.29	0.84	4.11	0.996 (0.993–0.998)	0.973	0.24	0.68

*Abbreviations:* *SD* Standard deviation, *ICC* Intraclass correlation coefficient, *SEM* Standard error of measurement, *MDC* Minimal change detectable, *CI* Confidence interval, *BARE* Barefoot, *VRFW3* Varus rearfoot wedge 3°, *VRFW5* Varus rearfoot wedge 5°, *VRFW8* Varus rearfoot wedge 8°

**Table 3:** The table shows the results in Newtons for each situation

Variables	BARE Mean (degrees) ± SD (95% CI)	VRFW3 Mean (degrees) ± SD (95% CI)	VRFW5 Mean (degrees) ± SD (95% CI)	VRFW8 Mean (degrees) ± SD (95% CI)	* P* Value BARE vs. VRFW3	* P* Value BARE vs. VRFW5	* P* Value BARE vs. VRFW8	* P* Value VRFW3 vs. VRFW5	* P* Value VRFW3 vs. VRFW8	* P* Value VRFW5 vs. VRFW8
Newtons(N)	19.62 ± 5.18 (9.38–29.9)	16.01 ± 4.10 (10.6–23.68)	14.42 ± 3.98 (6.83–22.23)	12.51 ± 4.12 (5.95–21.48)	0.001**	0.001**	0.001**	0.001**	0.001**	0.001**

*Abbreviations:* *SD* Standard deviation, *CI* Confidence interval, *BARE* Barefoot, *VRFW3* Varus rearfoot wedge 3°, *VRFW5* Varus rearfoot wedge 5°, *VRFW8* Varus rearfoot wedge 8°

*P* value = level of significance; *p* < 0.05 was considered statistically significant, and *p* < 0.001** was considered strongly statistically significant

## Discussion

This study aimed to assess the impact of VRFWs on the first MTPJ resistance during Jack’s Test. The key finding was that VRFWs led to a significant reduction in resistance to dorsiflexion of the proximal phalanx of the first MTPJ (*p* < 0.001). These results suggest that using VRFWs facilitates dorsiflexion movement with less resistance during Jack´s Test, thus providing a method for clinicians to quantify retrograde forces on the first MTPJ influenced by VRFW thickness. This insight into reduced force during Jack’s Test can aid clinicians in treatment decision-making. Notably, only the force for the movement was measured in this study, without determining joint stiffness. While these kinetic parameters are related, it is essential to acknowledge that comparative studies may not consistently employ the same parameters.

Various studies, including those by Kappel-Bargas [12] and Gatt [16], have explored the correlation between the first MTPJ range of motion during Jack’s Test and foot biomechanics during gait. Notably, these studies, unlike ours, focused solely on range of motion without considering joint resistance, and some conducted the test in partial weight-bearing conditions. In contrast, the studies by Sichting [13] and Halstead [17] found no significant relationship between Jack’s Test and range of motion limitations, although they did not account for joint resistance. Leow’s study [18] stands out as the first to quantify first MTPJ stiffness using a load cell and optical fibre with a fibre Bragg grating, establishing the reliability of this measurement in clinical settings. Our research aligns with Leow’s, as both studies measured the kinetic parameters of the first MTPJ.

Leow’s study differed by conducting a non-weight-bearing test, employing the stiffness parameter, while our work involved weight bearing and used the force parameter. In comparison to Moisan’s study [25], our barefoot results were lower by 19.62 ± 5.18 N, attributed to design variations in the devices. Sánchez-Gómez [31] used the same digital force gauge as ours, reporting slightly higher results (33.60 ± 1.36 N) in healthy subjects due to the absence of our lever and pulley system. Scherer [37] found no significant relationship between Jack’s Test and 3° VRFWs, but this study only focused on range of motion without recording first MTPJ resistance.

Our findings contrast with the results of Montenau [38], as we observed a notable reduction in first MTPJ resistance with VRFWs. Montenau’s study, involving VRFWs with orthoses during Jack’s Test, noted increased dorsiflexion of the first MTPJ, albeit not statistically significant. Few studies have compared Jack’s Test and orthopaedic devices in clinical settings. Our results offer clinicians a valuable tool for quantifying resistance during Jack’s Test and understanding its impact on foot tissue stress when using VRFWs.

### Limitations

To ensure accuracy, we checked the position of the digital force gauge head on the skin for each measurement during the test, considering that its displacement could alter the thrust position. To mitigate the impact of force application speed, we conducted up to three measurements in each condition, involving two different researchers. Recognizing that instability on a raised surface could introduce unintended variations, we took precautions to minimize such effects during sampling.

### Conclusions

The use of VRFWs has been observed to reduce dorsiflexion resistance in the proximal phalanx of the first MTPJ during Jack’s Test. Additionally, the digital force gauge has been proven to be a valid tool for conducting Jack’s Test, offering a reliable means of measurement.

## Data Availability

The datasets used and analysed during the current study are available from the corresponding author upon reasonable request.

## Abbreviations

MTPJ: Metatarsophalangeal joint
FPI: Foot Posture Index
VRFW: Varus rearfoot wedges
SD: Standard Deviation
ICC: Intraclass correlation coefficient
SEM: Standard error of measurement
MDC: Minimal change detectable
CI: Confidence Interval
