# Correction: The effect of varus rearfoot wedges on hallux dorsiflexion resistance

**DOI:** 10.1186/s12891-024-07280-w

**Published:** 2024-02-26

**Authors:** Álvaro Gómez‑Carrión, José Manuel Reguera-Medina, Ignacio Ayerra‑Andueza, Juan Francisco Cortés‑Morán, Alfonso Martínez‑Nova, Rubén Sánchez‑Gómez

**Affiliations:** 1 Nursing Department, Faculty of Nursing, Physiotherapy, and Podiatry, Universidad Complutense de Madrid, Madrid, 28040 Spain; 2 Clinical Sanipie, Utrera, Sevilla 41704 Spain; 3 Clinical Ayerra, Almeria, 04001 Spain; 4 Nursing Department, Universidad de Extremadura, Plasencia, 10600 Spain; 5 IdISSC, Institute for Health Research, Hospital Clínico San Carlos, Madrid, 28040 Spain

**Correction: BMC Musculoskelet Disord 25, 84 (2024)**

10.1186/s12891-024-07182-x

Following publication of the original article [1], the authors corrected the surname of the 2nd author to “Reguera-Medina” instead of “Medina-Reguera”.

The author group has been updated above and the original article [1] has been corrected.
